# Evolution of Immune Checkpoint Blockade in Metastatic NSCLC: A Narrative Review of Emerging Bispecific Antibodies and the Practical Challenges of Clinical Integration

**DOI:** 10.3390/cancers18040709

**Published:** 2026-02-22

**Authors:** Jin Hyoung Kang

**Affiliations:** Department of Medical Oncology, The Catholic University of Korea, Seoul St. Mary’s Hospital, Room 9213, 9th Floor, Annexed Building, 222, Seoul 06591, Republic of Korea; oncologykang@naver.com; Tel.: +82-2-2258-6546

**Keywords:** mNSCLC, immune checkpoint inhibitor, bispecific antibody, novel toxicity, PK mismatch, standard-of-care

## Abstract

**Background/Objectives**: Metastatic non-small cell lung cancer (mNSCLC) is challenged by toxicity, pharmacokinetic (PK) mismatch, and resistance inherent in current immune checkpoint inhibitor (ICI) regimens. Bispecific antibodies (BsAbs), which unify dual-target engagement within a single, structurally engineered molecule, offer a next-generation strategy to transcend these therapeutic ceilings. Their engineering formats—ranging from IgG-like full-size antibodies to non-IgG-like fragment-based or smaller scaffolds—are selected to further optimize target affinity and enhance therapeutic efficacy. **Methods**: This narrative review analyzes landmark trials establishing the current standard of care (SoC) and evaluates the mechanistic rationale and status of emerging BsAb, including dual checkpoint blockers and tumor microenvironment (TME)-modulating BsAbs, using the data from the latest early-phase clinical studies. **Results**: The review critically focuses on comparing the theoretical benefits of BsAbs, such as PK consistency and synergistic efficacy via affinity-controlled dual targeting, against the practical drawbacks of conventional combination therapies. Most importantly, it assesses the BsAbs’ potential to secure superior overall survival (OS) or progression-free survival (PFS) against established SoC and deeply reviews the clinical feasibility of managing their unique safety profiles. **Conclusions**: While BsAbs offer a potent, innovative approach to enhancing anti-tumor immunity, substantial hurdles remain for their widespread clinical integration. Key challenges include: (1) demonstrating clear survival benefits over high-efficacy SoCs in late-stage trials; (2) establishing guidelines for managing novel toxicity profiles; and (3) addressing logistical barriers related to complex manufacturing and high costs. Furthermore, the validation of predictive biomarkers is essential to guide the personalized application of BsAb-based immunotherapies.

## Simple Summary

Metastatic non-small cell lung cancer (mNSCLC) remains difficult to treat due to the toxic side effects, pharmacokinetic (PK) mismatch, and drug resistance in current combination regimens of immune checkpoint inhibitor (ICI). Bispecific antibodies (BsAbs) represent a novel approach designed to overcome these hurdles by simultaneously targeting two different pathways with a single molecule. By using diverse engineering formats through precise structural design, BsAbs can maximize synergistic efficacy, and minimize toxicity. The aim of the current review is to analyze the mechanistic rationale and current status of emerging BsAbs, and to evaluate whether BsAbs can outperform the current standard of care (SoC). Our findings suggest that while BsAbs offer theoretical advantages, they also face significant practical challenges, including the need to demonstrate superior survival rates in large-scale trials, manage unique side effects, high manufacturing costs, and validate personalized BsAb-based immunotherapy through predictive biomarkers.

## 1. Introduction

Metastatic non-small cell lung cancer (mNSCLC) constitutes a principal cause of cancer-related mortality globally, thereby representing a significant clinical burden and an imperative therapeutic void [1,2]. Historically, therapeutic intervention relied predominantly on platinum-based chemotherapy, which typically afforded only limited benefits regarding overall survival (OS). The subsequent introduction of targeted therapies provided patient-specific options for individuals harboring oncogenic drivers (e.g., Epidermal Growth Factor Receptor [EGFR], Anaplastic Lymphoma Kinase [ALK]); nevertheless, the clinical utility of these modalities is inherently constrained by the prevalence of such mutations and the inevitable emergence of acquired resistance mechanisms.

The treatment paradigm experienced a fundamental shift with the advent of immune checkpoint inhibitors (ICIs), primarily directed against the programmed cell death protein 1 (PD-1)/programmed death-ligand 1 (PD-L1) axis. ICIs have demonstrated durable anti-tumor responses, fundamentally altering the prognosis for numerous patients diagnosed with advanced NSCLC [3,4,5]. While ICIs have recently moved into neoadjuvant and adjuvant settings for early-stage disease, this review exclusively focuses on the therapeutic landscape and challenges in mNSCLC (Stage IV), where systemic resistance remains the primary barrier to cure. Following the established success of monotherapy in patients with high PD-L1 expression, combination strategies—including chemo-immunotherapy (e.g., KEYNOTE-189) and dual checkpoint blockade-based regimens (e.g., CheckMate 227, CheckMate 9LA)—have been instituted as the new standard of care (SoC), effectively broadening therapeutic efficacy across diverse patient populations [6,7,8].

Notwithstanding, these combination regimens are associated with considerable practical limitations in clinical practice. The concurrent administration of multiple agents frequently exacerbates high-grade toxicities; pharmacokinetic (PK) mismatch between the different agents can lead to suboptimal therapeutic windows; and a substantial subset of patients demonstrates primary or acquired resistance, often driven by the immunosuppressive nature of the tumor microenvironment (TME) [9,10]. In particular, the lack of selectivity in conventional therapies and the physiological barriers within the TME often impede effective drug delivery, further facilitating immune escape [11]. These persistent clinical hurdles necessitate the rigorous exploration of next-generation immunotherapeutic platforms capable of integrating synergistic mechanisms while simultaneously streamlining drug delivery and improving overall toxicity management.

Bispecific antibodies (BsAbs) emerge as a highly compelling therapeutic modality. These agents are engineered to simultaneously engage two distinct epitopes—such as dual immune checkpoints (e.g., PD-1 and Lymphocyte-Activation Gene 3 [LAG-3]) or an immune checkpoint combined with a TME modulator (e.g., PD-1 and Vascular Endothelial Growth Factor [VEGF])—and are thus posited to confer theoretical advantages, including consistent PK profiles, localized synergistic activity, and potentially mitigated systemic toxicity achieved through affinity optimization [12]. The therapeutic rationale for BsAbs lies in their ability to induce spatial synergy—such as physical bridging between T-cells and tumor cells—which enhances anti-tumor potency and selectivity compared to the simple co-administration of independent monoclonal antibodies.

Despite these bio-engineering breakthroughs, a critical research gap remains in translating molecular precision into actionable clinical strategies, particularly regarding treatment sequencing and novel toxicity management. The objectives of this narrative review are twofold: First, to critically evaluate the mechanistic and clinical rationale supporting BsAbs as a potentially superior alternative to current ICI combination strategies for mNSCLC. Second, and more crucially, to provide an evidence-based assessment of the potential of BsAbs to secure superior OS or progression-free survival (PFS) against the established SoC and to address the critical practical challenges pertaining to the clinical integration and management of novel toxicity profiles. By synthesizing mechanistic advantages with practical implementation strategies, we aim to provide a roadmap for the successful integration of BsAbs into the evolving oncology landscape. However, it is important to note the inherent limitations of this review, including its narrative approach, which lacks formal systematic protocolization, and the challenge of interpreting overall survival data that remain immature for many emerging candidates.

## 2. Materials and Methods

The current narrative review was developed through the clinical perspective of a medical oncologist with over four decades of experience in thoracic oncology, and is reported in accordance with the scale for the quality assessment of narrative review articles (SANRA) guidelines [13]. Although the review did not fully satisfy all SANRA criteria, particularly the third category regarding the description of the literature search, every effort was made to adhere to the guidelines as comprehensively as possible, where the Table S1 provides details of the checklist items [14,15,16,17,18,19,20,21,22,23,24,25,26,27,28,29,30,31,32]. Given the nature of a narrative review and the heterogeneity of study designs, interventions, and endpoints in the available literature, no systematic study selection process was planned or conducted. Instead, additional publications were identified through the use of “a snowball technique”, which is a clinically driven and historically grounded approach as follows:

(1) Key developments and pivotal trials were first mapped from expert field knowledge to ensure historical completeness and clinical relevance. The article incorporates landmark phase II–III trials and authoritative mechanistic reports.
(2) These reference points were then verified and expanded through targeted literature searches utilizing targeted explorations of PubMed and Scopus, which served as the primary databases.

This approach offers a purposefully identified and clinically curated synthesis, providing interpretive depth and practical applicability.

## 3. Results

### 3.1. The Era of ICI Monotherapy

The emergence of ICI monotherapy marked the initial revolutionary phase in the treatment of mNSCLC. ICI monotherapies introduced the possibility of durable, long-term survival, but clearly exposed limitations related to low overall response rates (ORR) in unselected populations and dependency on PD-L1 expression.

The foundation of this era rested on the blockade of the PD-1/PD-L1 pathway, thereby releasing the inhibitory signal on activated T-cells and reinstating anti-tumor immunity. The initial proof-of-concept for immune modulation was established by Ipilimumab, an anti-cytotoxic T-lymphocyte-associated protein 4 (CTLA-4) antibody, which demonstrated durable survival in advanced melanoma, albeit with substantial immune-related toxicity [33]. Subsequently, PD-1 inhibition emerged as a more versatile therapeutic option. Nivolumab showed impressive OS benefits versus docetaxel in both squamous and non-squamous NSCLC (CheckMate 017 and 057), demonstrating notable efficacy in squamous disease regardless of PD-L1 expression [3,4]. In contrast, Pembrolizumab adopted a distinct, biomarker-driven strategy. Through KEYNOTE-024 and KEYNOTE-010, it validated the hypothesis that high PD-L1 expression (tumor proportion score, TPS ≥ 50%) could identify the patients most likely to benefit, establishing single-agent checkpoint blockade as a standard first-line option for this biomarker-selected group [34,35]. These trials immediately underscored the critical importance of PD-L1 TPS as a predictive biomarker; while a clear OS benefit was achieved in the TPS ≥ 50% subgroup, efficacy in the TPS 1–49% range was often less compelling and subject to ongoing debate, highlighting a fundamental limitation in patient selection. Furthermore, PD-L1 inhibitors such as Atezolizumab (IMpower110) and Durvalumab also provided key efficacy data, establishing the class effect of PD-L1 blockade [36,37]. Durvalumab, while most recognized for its role in the Stage III consolidation setting (PACIFIC trial), fundamentally underscored the efficacy of PD-L1 blockade across different disease stages and further solidified the importance of ICI monotherapy mechanisms.

Despite the success of these first-generation PD-1/PD-L1 inhibitors, the clinical imperative to further optimize efficacy and extend the duration of activity persisted. Addressing this need, Tislelizumab emerged with specific engineering designed to overcome structural limitations of earlier agents [38]. Tislelizumab is an anti-PD-1 monoclonal antibody engineered with a modified Fc region to decrease binding to Fcγ receptors on macrophages, thereby reducing macrophage-mediated T-cell clearance—a limitation observed in first-generation PD-1 inhibitors [38]. Furthermore, Tislelizumab binds distinctively to PD-1, blocking over 99% of the PD-1/PD-L1 pathway with high target affinity and a slow dissociation rate, characteristics that lead to a significantly extended half-life [39]. These structural optimizations culminated in positive clinical outcomes, including a significant OS improvement for monotherapy in second and later lines through the RATIONALE-303 study [40].

Despite the breakthrough and the promise of durable disease control, the clinical impact of ICI monotherapy was constrained by two major issues. First, the ORR in the broader, unselected patient population often remained modest (typically ~15–20%). Second, the frequent occurrence of primary resistance (lack of initial response) and secondary/acquired resistance (disease progression after initial response) drove the therapeutic shift toward multimodal approaches. This necessity to overcome resistance and transform the highly immunosuppressive TME directly paved the way for the subsequent combination therapy era.

### 3.2. Combination Therapy Era

The limitations observed during the ICI monotherapy era—specifically low response rates in PD-L1-low/negative patients and the prevalence of primary resistance—drove the evolution toward combination strategies. ICI combination therapies successfully expanded efficacy irrespective of PD-L1 expression, establishing a new SoC, but concurrently introduced significant clinical issues such as amplified toxicities and inherent PK mismatch.

The combination of chemotherapy and ICI rapidly established itself as a cornerstone SoC, effectively maximizing clinical efficacy irrespective of PD-L1 expression by leveraging a strong mechanistic synergy. This synergy is rooted in the concept that chemotherapy induces immunogenic cell death (ICD), releasing neoantigens and danger-associated molecular patterns (DAMPs) that “prime” the immune system, thereby creating a “hotter” TME potentiated by checkpoint blockade [41]. This approach was validated by pivotal trials such as KEYNOTE-189 (Pembrolizumab + Pemetrexed/Platinum), IMpower150 (Atezolizumab + Bevacizumab + Chemotherapy) and RATIONALE-304 (Tislelizumab + Chemotherapy) for non-squamous mNSCLC [6,42], and KEYNOTE-407 (Pembrolizumab + Chemotherapy) as well as RATIONALE-307 (Tislelizumab + Chemotherapy) for squamous mNSCLC [43,44]. These trials demonstrated superior PFS and OS across all PD-L1 subgroups compared to chemotherapy alone, firmly establishing chemoimmunotherapy (chemo-IO) as the preferred first-line treatment for most advanced NSCLC patients. However, despite the enhanced efficacy, a critical drawback of this strategy is the intensification of toxicity. Data consistently revealed higher rates of Grade ≥ 3 adverse events (AEs), combining the inherent toxicities of chemotherapy (e.g., myelosuppression, fatigue) with immune-related adverse events (irAEs), complicating patient management [6,7,42].

In parallel, the dual blockade of PD-1 and CTLA-4 provided a complementary pathway for immune activation. This IO-IO strategy aims to overcome resistance by targeting both checkpoints: CTLA-4 inhibition enhances T-cell priming in lymph nodes, while PD-1 inhibition sustains the activation of CD8^+^ T-cells within the tumor site. The CheckMate 227 (Nivolumab + Ipilimumab) trial demonstrated the potential of this combination, crucially establishing a chemotherapy-free treatment option that demonstrated OS benefit in the patients with PD-L1 expression < 1%[8]. Building on this dual blockade strategy, the CheckMate 9LA trial introduced a refined strategy by combining dual checkpoint inhibition (Nivolumab plus Ipilimumab) with a limited course (two cycles) of chemotherapy [7]. This regimen aimed to mitigate the risk of early disease progression often seen with ICI monotherapy while establishing durable anti-tumor immunity through dual blockade, demonstrating a significant survival benefit compared to chemotherapy alone, especially for patients with PD-L1 < 1% [7]. Nevertheless, this regimen is associated with a broader and often more severe spectrum of irAEs compared to monotherapy, reflecting the potent and sometimes indiscriminate nature of dual immune activation [8].

Despite these significant gains in efficacy, conventional combination regimens ultimately exposed clear pharmacological and clinical limitations. The most notable problem is PK mismatch and logistical complexity. The co-administration of two distinct therapeutic antibodies or an antibody and chemotherapy introduces therapeutic challenges, as the agents possess different half-lives and distribution profiles [45]. This difference makes it difficult to maintain optimal drug concentration ratios for desired sufficient synergistic effect throughout the entire treatment cycle. Furthermore, conventional combinations remain limited in their ability to fully overcome intrinsic resistance mechanisms driven by the highly immunosuppressive TME [46]. For instance, the highly immunosuppressive TME often correlates with PD-L1 expression levels below 1%, making it a challenging population for single-agent ICI. Moreover, the low penetration of therapeutic antibodies across the blood–brain barrier (BBB) means that the high incidence of central nervous system (CNS) metastases in mNSCLC represents another significant and often unaddressed clinical limitation for current combination regimens. Factors such as tumor hypoxia, dense fibrosis (driven by Transforming Growth Factor-β [TGF-β]), and aberrant tumor vasculature (driven by VEGF) create a physical and chemical barrier that excludes effector T-cells, rendering checkpoint blockade ineffective [12,47]. This necessity to overcome complex immunosuppressive TME and structural PK issues directly drives the shift toward next-generation strategies (Table 1).

### 3.3. Emerging Strategies: Bispecific Antibodies

The emergence of BsAbs as a next-generation strategy is driven by the need to overcome the PK and toxicity limitations of conventional combination therapy by integrating complex mechanisms within a single molecular entity.

The fundamental advantage of the BsAb platform lies in its ability to simultaneously engage two distinct targets while maintaining a single PK profile [57]. This structural homogeneity addresses the PK mismatch problem seen in co-administered chemotherapy-free drug combinations and simplifies administration logistics [57]. BsAb design utilizes various engineering techniques, categorized generally into immunoglobulin G (IgG)-like (full-size antibodies), which typically maintain the standard Y-shape and full serum half-life, and Non-IgG-like structures (fragment-based or smaller scaffolds), which offer increased flexibility but often have shorter half-lives [58]. The choice of design significantly impacts the drug’s stability, half-life, and potential immunogenicity, factors that are critical for long-term clinical efficacy and safety.

Crucially, the engineering of BsAbs extends beyond the broad categorization of IgG-like versus Non-IgG-like structures to specific considerations of valency and geometry. The configuration of binding domains—whether in a symmetric 2:2 format (two binding sites for each target) or an asymmetric 1:1 format—fundamentally dictates the drug’s avidity, tissue penetration, and ability to crosslink cells. For instance, a 1:1 monovalent design may be preferable to prevent target receptor internalization or to reduce toxicity by lowering avidity for healthy tissues, whereas a 2:2 format might be utilized to maximize receptor clustering and signal transduction in the tumor [59].

Beyond the binding domains (Fabs), Fc-region engineering plays a pivotal role in optimizing the therapeutic window. While a wild-type Fc region provides stability, targeted modifications—such as “knobs-into-holes” for heavy chain heterodimerization [60], LALA (L234A/L235A) mutations to eliminate unwanted effector functions (e.g., Antibody-Dependent Cellular Cytotoxicity, ADCC/Antibody-Dependent Cellular Phagocytosis, ADCP) that could deplete essential T-cell populations [61,62], and mutations to tune FcRn (neonatal Fc receptor) binding—are essential for ensuring a consistent half-life. This comprehensive molecular tuning ensures the stoichiometric co-engagement of both targets, ensuring that the synergistic potential of dual-targeting is not compromised by the staggered drug clearance often observed in free combinations.

**Figure 1:**
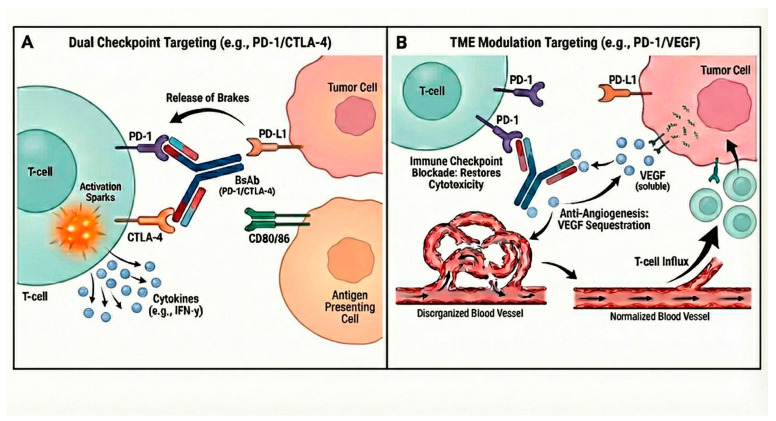
Mechanistic rationale of emerging bispecific antibodies in metastatic non-small cell lung cancer. (**A**) Dual Checkpoint Targeting: Bispecific antibodies (e.g., targeting PD-1 and CTLA-4) bind simultaneously to two distinct inhibitory receptors on T-cells. This dual blockade lowers the threshold for T-cell activation more potently than single-agent therapy, enhancing anti-tumor immune responses. (**B**) Tumor Microenvironment (TME) Modulation: Bispecific antibodies (PD-1/VEGF) utilize a “cooperative binding” mechanism. The anti-PD-1 domain anchors the drug to T-cells within the tumor, while the anti-VEGF domain sequesters soluble VEGF. This localized action promotes vascular normalization and increases T-cell infiltration (spatial synergy) while mitigating systemic toxicity associated with peripheral VEGF blockade.

BsAb development is currently exploring two main mechanistic approaches to enhance anti-tumor immunity (Figure 1). The first approach involves dual checkpoint targeting, aiming to maximize T-cell activation by simultaneously blocking two inhibitory pathways, such as PD-1xCTLA-4 (e.g., MEDI5752), PD-1xLAG-3 (e.g., Tebotelimab), or PD-1xT-cell Immunoreceptor with Ig and inhibitory motif (ITIM) domains, such as TIGIT (e.g., AZD2936). The second, and increasingly sophisticated, approach is TME modulation targeting. This strategy pairs an ICI target with a factor that contributes to the immunosuppressive TME, such as PD-1xVEGF (e.g., Ivonesimab [AK112]) or PD-L1xTGF-β (e.g., Bintrafusp alfa) [63]. This TME-focused strategy offers mechanistic depth: for example, VEGF targeting aims to normalize the abnormal tumor vasculature, thereby enhancing T-cell infiltration [64]. Similarly, Bintrafusp alfa employs a unique “trap” mechanism where the TGF-β receptor domain fused to the anti-PD-L1 antibody sequesters soluble TGF-β. This action seeks to dismantle the physical fibrotic barrier and immune-excluded phenotype driven by TGF-β, thereby facilitating effector T-cell infiltration into the tumor core where the concurrent PD-L1 blockade can exert its effect [65].

The rapidly expanding pipeline of BsAbs reflects the expeditiousness of translating these theoretical advantages into clinical reality. These candidates are swiftly progressing through early-phase trials, suggesting that BsAbs are poised to challenge the current paradigm of ICI-based combination therapy (Table 2 and Table S1).

### 3.4. Mechanistic and Pharmacokinetic Advantages of Bispecific Antibodies over Biologic Combinations

BsAbs offer compelling theoretical advantages over conventional biologic combination therapies by providing consistent PK profiles as single agents, maximizing synergistic efficacy, and minimizing toxicity through precise affinity optimization. The pharmacological superiority of BsAbs over co-administered monoclonal antibodies (mAbs) is fourfold:

First, regarding pharmacokinetic consistency, clinicians often grapple with distinct half-lives and distribution profiles, leading to temporal windows where the optimal synergistic ratio of the drugs is lost [72]. A BsAb, being a single molecule, ensures that both targets are engaged simultaneously and proportionally throughout the dosing interval, thereby maintaining consistent therapeutic pressure [73]. As detailed in the preceding section, the integration of targeted mutation within the Fc domain (e.g., LALA mutations) allows for the precise modulation of serum half-life (e.g., via FcRn binding mutations) and the elimination of off-target effector functions, further optimizing the PK profile beyond simple unification [73]. Additionally, genetic engineering to control glycosylation patterns offers a strategic avenue to reduce drug clearance and mitigate immunogenicity, ensuring more stable therapeutic exposure [74].

Second, BsAbs streamline clinical development by bypassing extensive, time-consuming, and costly dose-finding studies to determine the optimal schedule and ratio for two independent agents [72]. This efficiency not only accelerates the drug development timeline but also reduces the logistical and financial burdens associated with establishing a novel therapeutic regimen.

Third, BsAbs enable sophisticated toxicity management through “affinity tuning,” which is unattainable with separate agents. Unlike the indiscriminate systemic activation often seen with dual checkpoint blockade (e.g., anti-CTLA-4 plus anti-PD-1), BsAbs can be engineered with differential affinities for their targets [72]. For instance, a BsAb can be designed to bind with low affinity to a ubiquitously expressed target (e.g., CTLA-4) and high affinity to a tumor-enriched target (e.g., PD-1). This ensures that the drug preferentially accumulates and becomes active only in the TME where both antigens are co-expressed, thereby sparing normal tissues from autoimmune-like toxicities. This “avidity-driven” binding allows for a wider therapeutic window compared to the co-administration of high-affinity monospecific antibodies [75]. Moreover, emerging clinical evidence suggests that BsAbs may also offer advantages in tolerability and drug–drug interaction profiles compared with classical ICI regimens; the more localized and avidity-dependent activity of BsAbs may mitigate systemic toxicities, potentially supporting more consistent therapeutic outcomes [76,77].

Finally, BsAbs facilitate enhanced localized synergy that is often unattainable with separate agents. By physically bridging two distinct receptors on the same cell (cis-binding) or on adjacent cells (trans-binding), BsAbs can force specific immunological synapses or receptor clustering events that drive potent T-cell activation [78]. In certain configurations, this may even allow for major histocompatibility complex (MHC)-independent activation of T-cells through direct receptor cross-linking, effectively bypassing some of the standard constraints of antigen presentation and amplifying anti-tumor efficacy precisely at the tumor site [79]. This localized bridging not only amplifies the signaling magnitude but also directs the immune response precisely to the tumor site, effectively converting a “cold” TME into an immunologically “hot” state more efficiently than the stochastic interactions inherent to conventional combination therapies [78].

### 3.5. Current Evidence: Efficacy and Novel Toxicity

While BsAbs possess a strong mechanistic rationale, their clinical integration relies heavily on demonstrating competitive efficacy against the current high bar of SoC chemo-immunotherapy and effectively managing novel toxicity profiles.

#### 3.5.1. Early-Phase Efficacy Compared to Standard-of-Care

The initial clinical landscape of BsAbs in mNSCLC is characterized by promising signals of efficacy, particularly in early-phase trials. Several BsAb candidates targeting dual checkpoints (e.g., PD-1xCTLA-4, PD-1xLAG-3) have demonstrated encouraging ORR and disease control rates (DCR). However, interpreting these “promising” results requires a critical contextualization against the established efficacy of current first-line SoC regimens. For instance, the KEYNOTE-189 regimen (pembrolizumab plus chemotherapy) set a robust benchmark with an ORR of approximately 48% and significant survival benefits [80]. Consequently, for a BsAb to displace or even complement this regimen, it must demonstrate not merely “clinical activity” but a clear superiority in ORR, durability of response (DOR), or a significantly improved safety profile. Current early-phase data for many BsAbs often show ORRs in the range of 30–45% in heterogenous populations, suggesting that while they are active, they do not yet uniformly outperform the chemo-IO benchmark in unselected patients. This discrepancy underscores the urgent need for biomarker-driven patient selection to identify subgroups where BsAbs may offer a definitive advantage over current standards.

#### 3.5.2. Clinical Breakthroughs in TME Modulation: Focus on PD-1xVEGF Bispecifics

Among the emerging strategies, TME-modulating BsAbs, particularly those targeting PD-1xVEGF, have generated arguably the most compelling clinical data to date. While several candidates are in development, clinical evidence for PD-1xVEGF BsAbs represents a paradigm shift by addressing tumor angiogenesis and immune suppression simultaneously. Lead molecules in this class, such as Ivonesimab (AK112), have shown impressive phase 2 efficacy, with ORRs reportedly exceeding 50% in certain cohorts, including those with PD-L1-positive tumors and, notably, in patients with EGFR-mutated mNSCLC who have progressed on EGFR tyrosine kinase inhibitors (TKIs) [81]. This latter finding is clinically significant as it suggests that the simultaneous blockade of VEGF (normalizing tumor vasculature) and PD-1 can overcome the immunologically “cold” and hypoxic TME characteristic of TKI-resistant disease—a setting where traditional ICI monotherapy or combinations have historically struggled. These results position TME-modulating BsAbs as a potentially superior strategy for specifically difficult-to-treat IO-resistant patient populations.

Building on these phase 2 signals, subsequent phase 3 results (e.g., HARMONi-2 and HARMONi-A) have confirmed the high potency of this platform, with Ivonesimab demonstrating a statistically significant PFS improvement over established standards [66]. For instance, while the HARMONi-A trial reported a significant PFS benefit (HR 0.46), confirming the potential for long-term survival in biomarker-selected cohorts, global OS results for unselected first-line patients are still maturing [67].

The structural design of PD-1xVEGF BsAbs offers a compelling mechanistic rationale to address the challenges encountered in previous combination trials. For instance, the IMpower150 trial (atezolizumab, bevacizumab, and chemotherapy) demonstrated a PFS benefit but faced difficulties in translating this into a definitive OS advantage, potentially because the independent biodistribution of separate agents limits the spatial and temporal synchronization required for optimal synergistic vascular normalization and immune activation. In contrast, the bispecific format utilizes a “cooperative binding” mechanism: the anti-PD-1 domain serves as an anchor, concentrating the anti-VEGF moiety within the TME [82]. This “cis-targeting” capability is hypothesized to maximize local vascular normalization and immune cell infiltration while mitigating systemic off-target effects [83]. Such a refined therapeutic index suggests that the bispecific format may be better positioned to overcome the clinical hurdles that have limited the success of traditional anti-VEGF/ICI combinations.

However, interpreting these clinical breakthroughs requires a nuanced understanding of the ongoing maturation of OS data. While significant OS benefits were observed in earlier Chinese trials like HARMONi-A, results from more recent Western cohorts are still evolving and have yet to reach statistical maturity. Although preliminary updates show a positive trend as follow-up time increases, current data are not yet conclusive. This temporal lag remains a critical challenge in determining whether the mechanistic advantages of TME-modulating BsAbs will ultimately translate into a definitive, universal survival benefit, as discussed in detail later (Section 3.6.4).

#### 3.5.3. Divergent Toxicity Profiles and Management Challenges

The clinical introduction of BsAbs brings forth a complex safety landscape that diverges from the familiar irAEs of monoclonal ICIs, encompassing both conventional and novel side effects. The toxicity profile of BsAbs generally is generally categorized based on their mechanistic targets. Dual checkpoint BsAbs (e.g., PD-1xCTLA-4) tend to exhibit a spectrum of common irAEs similar to, but occasionally more frequent than, their separate combination counterparts, necessitating stringent warnings and vigilant monitoring for high grade immune-mediated colitis or hepatitis [84,85]. Conversely, TME-modulating BsAbs introduce target-specific toxicities; for example, PD-1xVEGF bispecifics are associated with VEGF-related adverse events such as hypertension, proteinuria, and bleeding events, requiring oncologists to integrate cardiovascular monitoring into routine immune-oncology care [86]. Crucially, the toxicity profile of BsAbs represents a qualitative shift from the hematologic and gastrointestinal burdens typical of standard chemo-immunotherapy. While chemo-immunotherapy is characterized by myelosuppression (e.g., neutropenia) and chemotherapy-induced nausea, BsAbs substitute these chemotherapy-driven systemic toxicities with a higher intensity of immune-mediated effects or specific off-tumor/on-target signals, such as VEGF-driven hypertension or transient cytokine release.

Furthermore, a distinct and novel toxicity concern with BsAbs is the potential for Cytokine Release Syndrome (CRS) and infusion-related reactions (IRRs). Although generally less severe than those observed with Blinatumomab used in hematologic malignancies [87,88] or Tarlatamab (DLL3-targeted BiTE), which was recently approved for previously treated small cell lung cancer (SCLC) [89], the occurrence of low-grade CRS in solid tumor trials mandates new management protocols [90]. To effectively navigate these challenges, clinicians must be equipped to distinguish acute systemic inflammatory re-sponses from standard hypersensitivity or irAEs. Additionally, integrating the Immune-Response Evaluation Criteria in Solid Tumors (iRECIST) criteria into clinical assessment frameworks allows for the differentiation of true progression from immune-related flares or delayed responses [91]. Finally, while contraindications are still being defined in evolving trials, BsAbs are generally avoided in patients with severe active autoimmune diseases or significant baseline organ dysfunction to prevent exacerbated inflammatory responses. The successful clinical adoption of BsAbs will depend not only on their efficacy but also on the establishment and/or implementation of such comprehensive criteria that address these novel safety signals, ensuring that the therapeutic index remains favorable compared to established chemo-immunotherapy regimens.

### 3.6. Translating Promise into Practice: Challenges of BsAbs Integration

The transition of BsAbs from promising biological constructs to standard clinical practice faces significant hurdles, including high-profile trial failures, the necessity of proving superiority over established combinations, and the challenge of overcoming organ-specific resistance mechanisms.

#### 3.6.1. Biological Rationale vs. Reality in Clinical Trials: Lessons from Failure

Despite the elegant biological rationale of “bifunctional targeting”, the translation to clinical success is far from guaranteed. A prominent cautionary tale is Bintrafusp alfa (a bifunctional fusion protein targeting PD-L1xTGF-β), which failed to meet its primary endpoint in the phase 3 INTR@PID Lung 037 trial against pembrolizumab in PD-L1-high mNSCLC [92]. This failure highlights critical discrepancies between mechanistic theory and clinical reality. The “sink effect,” in which the drug is sequestered by ubiquitous circulating TGF-β and platelet-bound fraction before reaching the tumor, likely compromised the effective concentration within the TME [93]. Furthermore, the heterogeneity of TGF-β expression within the tumor stroma suggests that a “one-size-fits-all” approach may be insufficient without precise biomarker selection [93]. These challenges are not unique to bintrafusp alfa; other bispecific constructs, such as certain CEA-targeted T-cell bispecific (CEA-TCB) agents or early-generation PD-1xCTLA-4 bispecifics, have also encountered narrow therapeutic windows or limited efficacy compared to combination therapies [94]. This underscores that mere physical tethering of two active agents does not automatically translate to clinical synergy if PK biodistribution and target biology are not perfectly aligned. Beyond tumor-intrinsic biology, emerging evidence indicates that these discrepancies between mechanistic promise and clinical reality may also arise from patient-level modifiers, including concomitant medications [77]. To mitigate these risks, future trials should integrate dynamic PK/PD modeling and quantitative systems pharmacology to predict optimal target occupancy and ‘sink’ saturation levels prior to patient enrollment.

#### 3.6.2. The Gap Between BsAbs and Validated Combination Success

A critical benchmark for BsAbs is the long-term success of dual checkpoint inhibition seen with Nivolumab plus Ipilimumab (CheckMate 227) [8]. This combination has demonstrated durable 5-year OS benefits and is validated by extensive real-world evidence (RWE), confirming that simultaneous PD-1 and CTLA-4 blockade drives profound immune memory [95]. To date, dual-checkpoint targeting BsAbs have yet to unequivocally demonstrate this level of robust, long-term efficacy or superiority over the Nivo + Ipi regimen. This gap underscores a crucial distinction between “challenges in structural design” and “limitations inherent to the biological target” For dual-checkpoint BsAbs targeting established pairs like PD-1xCTLA-4, the challenge is primarily one of protein engineering—optimizing valence, geometry, and Fc effector functions to replicate or exceed the known synergistic potential of the separate antibodies. However, the failure of BsAbs targeting novel pairs, such as PD-1xLAG-3 or PD-1xTIM-3, to demonstrate significant efficacy in mNSCLC may reflect an inherent limitation of the biological targets themselves in this specific indication, rather than a shortcoming of the bispecific scaffold [96]. Practical solutions to bridge this gap involve the development of composite biomarkers that go beyond single-target expression, utilizing multi-omic profiling to identify specific patient subsets whose TME is uniquely driven by these secondary pathways.

#### 3.6.3. Addressing Organ-Specific Resistance: The BBB and Liver Metastasis

For BsAbs to become a transformative SoC, they must effectively address spatial resistance mechanisms in difficult-to-treat metastatic sites. Brain metastases present a formidable obstacle due to the BBB. However, in the context of established intracranial lesions, the blood–tumor barrier (BTB) becomes the primary interface. Although the BTB is structurally more permeable than the BBB, this aberrant “leakiness” paradoxically results in chronically elevated interstitial fluid pressure (IFP), which creates a physical barrier to the convective transport of large macromolecules like antibodies. By normalizing the aberrant tumor vasculature—a concept well-established in anti-angiogenic therapy—VEGF-targeting BsAbs such as Ivonesimab can significantly reduce IFP and upregulate endothelial adhesion molecules. This dual action facilitates the deeper penetration of therapeutics and the infiltration of effector T-cells across the BTB into CNS lesions [97]. Clinical validation of this mechanism was recently highlighted in the HARMONi-2 trial, where Ivonesimab monotherapy demonstrated consistent activity in patients with baseline brain metastases, reinforcing its potential to overcome spatial resistance [98].

Similarly, liver metastases represent an immunologically tolerant niche characterized by the accumulation of myeloid-derived suppressor cells (MDSCs) and T-cell exclusion. Recent insights into “systemic T-cell siphoning” suggest that hepatic involvement acts as a functional sink, sequestering and exhausting tumor-specific T-cells from the systemic circulation, thereby blunting the efficacy of monoclonal ICIs [62,99].TME-targeting BsAbs are uniquely positioned to reverse this phenomenon. By neutralizing pro-tumorigenic cytokines (e.g., TGF-β) and remodeling the extracellular matrix, these agents can dismantle the MDSC-rich stroma and restore the hepatic immune landscape. This reversal of the siphoning effect potentially allows for the expansion of the systemic T-cell pool, addressing one of the most significant clinical hurdles in mNSCLC [63,100]. The integration of radiomics and functional imaging (e.g., PET/MRI) will be essential to practically monitor TME remodeling and guide the timing of BsAb administration.

#### 3.6.4. Ethnic Heterogeneity and the Necessity of Global Validation

A significant proportion of the current clinical evidence for next-generation BsAbs originates from trials conducted primarily within the Chinese population, such as the HARMONi-2 and HARMONi-A studies. This regional concentration presents a challenge regarding the universal applicability of findings due to a combination of biological and logistical factors. Epidemiological disparities plays a critical role, as EGFR-mutated NSCLC—which is significantly more prevalent in East Asian populations (~40–50%) compared to Western cohorts (~10–15%)—exhibits distinct tumor microenvironments and angiogenic profiles. The VEGF pathway, targeted by antibodies like Ivonesimab, appears particularly influential in EGFR-driven tumorigenesis, which may explain the more immediate and pronounced OS benefits observed in Asian populations. However, it is important to note that definitive conclusions regarding long-term survival remain premature; while PFS data are robust, mature OS results from non-Asian cohorts are still evolving and have yet to demonstrate a consistent, statistically significant advantage in diverse global populations.

Furthermore, differences in regional SoC and the availability of post-progression therapies profoundly influence survival outcomes. In Western medical settings, the higher availability of advanced subsequent therapies, such as antibody-drug conjugates (ADCs) and fourth-generation TKIs, can introduce a significant ‘cross-over effect.’ As control group patients receive these potent later-line treatments, the survival gap between the investigational agent and the control is often diluted, complicating the interpretation of definitive OS gains. Additionally, the earlier activation in Chinese trials has provided higher data maturity compared to Western cohorts, although recent updates suggest survival metrics in the West are beginning to align with these positive trends as observation time accumulates. Consequently, global multi-regional trials are essential to validate these breakthroughs across diverse ethnic groups and establish a new global standard of care.

#### 3.6.5. Biomarker-Driven Selection and Integration Feasibility

The “all-comers” approach is increasingly untenable for BsAbs. Maximizing clinical benefit requires a biomarker-driven strategy that dictates the choice of BsAb format. Drawing from the paradigm established by the CheckMate 9LA and 227 trials, where dual checkpoint blockade demonstrated its most significant incremental gains in patients with PD-L1-low or negative tumors (<1%), dual checkpoint BsAbs (e.g., PD-1xCTLA-4) may be prioritized for these “cold” tumor populations, where conventional monotherapy often yields suboptimal responses [7,8]. In contrast, TME-modulating BsAbs (e.g., PD-1xVEGF) might be more specifically suited for tumors characterized by high vascularization or fibrotic “immune-excluded” phenotypes, potentially independent of PD-L1 status, by resolving the spatial barriers to immune cell infiltration [83]. However, defining these phenotypes through objective metrics remains challenging, as current single-analyte biomarkers like PD-L1 often fail to capture the dynamic complexity of the TME. Moving forward, the integration of multiplexed assays or transcriptomic signatures will be essential to transition from empirical use to precision patient stratification, ensuring that the therapeutic potential of BsAbs is matched with the appropriate biological context.

Beyond clinical considerations, the feasibility of integration extends beyond clinical efficacy to manufacturing and economics. The production of BsAbs involves complex genetic engineering and purification processes, leading to a significantly higher Cost of Goods (COGS) compared to monoclonal antibodies [101]. This economic reality poses a risk to global access and adoption. Unless these manufacturing complexities are addressed to achieve cost efficiencies, the widespread integration of BsAbs into the SoC will remain a substantial logistical challenge, especially in resource-constrained settings. Adopting continuous manufacturing platforms and modular “plug-and-play” bispecific scaffolds could substantially reduce COGS and improve global accessibility.

## 4. Conclusions and Future Directions

The evolution of immunotherapy in mNSCLC—from the initial breakthrough of monotherapy to the current standard of multimodal combinations—reflects a relentless pursuit to overcome therapeutic resistance and improve patient survival. Bispecific antibodies (BsAbs) represent the next logical step in this trajectory, offering a structural innovation designed to resolve the PK inconsistencies and toxicity limitations of conventional regimens, including the neutralization of imbalanced target-mediated drug disposition (TMDD) that often complicates free-drug combinations.

The introduction of ICIs fundamentally rewrote the treatment paradigm for mNSCLC, offering the first real hope for long-term survival in advanced disease. However, as this review has delineated, the transition from monotherapy to combination strategies, while expanding efficacy, has introduced new challenges: increased toxicity burden, logistical complexity, and the persistence of resistance driven by a hostile TME. BsAbs have emerged as a promising solution to these specific hurdles, providing a platform that ensures simultaneous target engagement, PK profiles, and the potential for safer, affinity-tuned immune activation.

Nevertheless, the path to establishing BsAbs as a new Standard of Care (SoC) is fraught with practical challenges. The clinical reality, underscored by high-profile trial failures, dictates that biological rationale alone is insufficient. To displace the current high-efficacy chemo-immunotherapy benchmarks, BsAbs must demonstrate a definitive OS benefit in large-scale, head-to-head randomized trials. Furthermore, the successful integration of these agents requires the oncology community to adapt to novel safety signals, such as CRS, and to address the significant economic and manufacturing barriers that threaten global patient access.

Looking ahead, the ultimate goal remains the realization of truly personalized immunotherapy. BsAbs are unlikely to be the final destination but rather a sophisticated backbone for future combinations. The next frontier involves integrating BsAbs with other potent modalities to dismantle residual resistance mechanisms. For instance, pairing BsAbs with antibody-drug conjugates (ADCs) could deliver cytotoxic payloads to stimulate immunogenic cell death in “cold” tumors, effectively fostering an ‘in situ vaccination effect’ that expands the repertoire of tumor-specific T-cells available for the BsAb to engage.

With the continuous influx of novel agents, including BsAbs, ADCs, and other modalities, clinicians face the growing complexity of determining the optimal ‘line-of-therapy’ strategy. Establishing a clear treatment sequence—specifically identifying the most effective salvage therapies after front-line BsAb exposure—is paramount to maximizing cumulative survival benefit. Ultimately, while further validation in large clinical trials is remains essential, the future of mNSCLC treatment lies in the strategic, biomarker-driven application of these advanced tools to tailor therapy to the unique immune architecture of each patient’s tumor.

## Figures and Tables

**Table 1:** Summary of Pivotal Clinical Trials for PD-1/L1 Inhibitors in Advanced or Metastatic NSCLC (Phase III trials).

Molecule (Reference)	Study Name	Study Design		Primary Endpoint HR [95% CI]	AE ^a^ Tx vs. Ctrl	Remarks
		*N*	Tx vs. Ctrl			
Nivolumab [3,4,7,8]	CheckMate-017	272	Nivo vs. Doce	OS: 0.59 [0.44–0.79]	7% vs. 55%	OS benefits over SOC regardless of PD-L1, SQ NSCLC
	CheckMate-057	582	Nivo vs. Doce	OS: 0.73 [96% CI, 0.59–0.89]	10% vs. 54%	OS benefits with increasing PD-L1, NSQ NSCLC
	CheckMate-227	1189	Tx A: Nivo + Ipi vs. Tx B: Nivo (PD-L1 ≥ 1%), Nivo + Chemo (PD-L1 < 1%) vs. Chemo	PD-L1 < 1%: OS: 0.62 [0.49–0.79]	32% vs. 36%	OS benefits among PD-L1 < 1%
	CheckMate-9LA	719	Nivo + Ipi + Chemo vs. Chemo	OS: 0.66 [0.55–0.80]	47% vs. 38%	Dual-pathway for recruiting T cells, effective in PD-L1 < 1% patients
Pembrolizumab [5,6,43]	KEYNOTE-024	306	Pembro vs. Chemo	OS: 0.60 [0.41–0.89]; PFS: 0.50 [0.37–0.68]	27% vs. 53%	Effective in PD-L1 ≥ 50% patients, the first monotherapy with pembro
	KEYNOTE-189	616	Pembro + Chemo vs. Placebo + Chemo	PFS: 0.52 [0.43–0.64]	67% vs. 66%	PFS benefits regardless of PD-L1, NSQ NSCLC
	KEYNOTE-407	559	Pembro + Chemo vs. Placebo + Chemo	OS: 0.64 [0.49–0.85]; [42,48–50PFS: 0.56 [0.45–0.70]	11% vs. 3%	Pembro-based combination therapy extended to SQ NSCLC
Atezolizumab [42,48,49,50]	OAK	1225	Atezo vs. Doce	OS: 0.73 [0.62–0.87]	37% vs. 54%	OS benefits with all PD-L1
	IMPower-150	1202	Tx A: Atezo + Chemo vs. Tx B: Atezo + Bevaci vs. Bevaci + Chemo	OS: 0.78 [0.64–0.96]	59% (tx B) vs. 50% (ctrl)	Improved PFS with liver meta and KRAS mutations
	IMPower-130	724	Atezo + Chemo vs. Chemo	OS: 0.79 [0.64–0.98]; PFS: 0.64 [0.54–0.77]	75% vs. 60%	OS benefits in patients w/o genetic mutations, NSQ NSCLC
	IMPower-110	572	Atezo + Chemo vs. Chemo	OS: 0.59 [0.40–0.89]	34% vs. 57%	The first PD-L1 inhibitor, effective in patients with high PD-L1
Durvalumab [51]	POSEIDON	1013	Tx A: Durva + Tremeli + Chemo vs. Tx B: Durva + Chemo vs. Chemo	Tx A: OS: 0.77 [0.65–0.92]; Tx B: OS: 0.86 [0.72–1.02]	52% (tx A) vs. 45% (tx B) vs. 44% (ctrl)	Proved the effect of triplet combo (PD-L1 + CTLA-4 + chemo)
Tislelizumab [44,52]	RATIONALE-304	332	Tisl + Chemo vs. Chemo	PFS: 0.65 [0.46–0.90]	Specified ^c^	Prolonged PFS, strong OS benefit in PD-L1 ≥ 50% subgroup, NSQ NSCLC
	RATIONALE-307	355	Tx A: Tisl + Chemo vs. Tx B: Tisl + Chemo ^b^ vs. Chemo	Tx A: PFS: 0.52 [0.37–0.74]; Tx B: PFS: 0.48 [0.34- 0.68]	Specified ^d^	Prolonged PFS where long-term follow-up indicates OS benefit, SQ NSCLC, approved in China, EMA, LATAM, and MENA
	RATIONALE-303	805	Tisl vs. Doce	OS: 0.64 [0.53–0.78]	Specified ^e^	Long-term OS benefits regardless of PD-L1 expression
Sugemalimab [53]	GEMSTONE-302	479	Sugemali + Chemo vs. Placebo + Chemo	PFS: 0.48 [0.39–0.61]	Specified	Approved in China and EMA
Cemiplimab [54,55,56]	EMPOWER-Lung1	712	Cemi vs. Chemo	OS: 0.57 [0.46–0.71]; PFS: 0.51 [0.42–0.62]	18% vs. 40%	Monotherapy with cemi in patients with PD-L1 ≥ 50%
	EMPOWER- Lung3	466	Cemi + Chemo vs. Placebo + Chemo	OS: 0.65 [0.51–0.82]; PFS: 0.55 [0.44–0.68]	49% vs. 33%	Benefit of combination therapy, across PD-L1 levels

Abbreviations: atezo, atezolizumab; bevaci, bevacizumab; cemi, cemiplimab; chemo, chemotherapy; CI, confidence interval; CTLA-4, Cytotoxic T-Lymphocyte Associated Protein 4; ctrl, control; doce, docetaxel; durva, durvalumab; EMA, European Medicines Agency; HR, hazard ratio; ipi, ipilimumab; KRAS, kirsten rat sarcoma viral oncogene homolog; LATAM, Latin America; MENA, Middle East and North Africa; nivo, nivolumab; NSCLC, non-small cell lung cancer; NSQ, non-squamous; OS, overall survival; pembro, pembrolizumab; PD-L1, Programmed Death-Ligand 1; PFS, progression-free survival; SOC, standard of care; SQ, squamous; tisl, tislelizumab; tremeli, tremelimumab; tx, treatment. ^a^ AE includes overall AEs of grade 3 or higher. ^b^ Chemotherapy regimens for Tx A and Tx B differ, respectively, paclitaxel (175 mg/m^2^, day 1) and carboplatin (area under the concentration of 5, day 1) for tx A, and nab-paclitaxel (100 mg/m^2^, days 1, 8, and 15) and carboplatin for tx B. ^c^ Overall AEs were not reported, the most common grade of 3 or greater AE was neutropenia (45% vs. 36%). ^d^ Overall AEs were not reported, the most common grade of 3 or greater AE was decreased neutrophil levels (52% vs. 46% vs. 45%). ^e^ Overall AEs were not reported, the most common grade of 3 or greater AE was pneumonia (8% vs. 9%).

**Table 2:** Bispecific antibody development in NSCLC (Phase II and III trials status).

Bispecific Antibody (Targets) [Reference]	Study	Phase	Eligibility	Intervention	Primary Endpoint	Interim Results: HR [95% CI], ORR/DCR (%, [95% CI])
Ivonescimab (PD-1 X VEGF) [64,65,66,67,68]	HARMONi-2	III, active, not recruiting	Stage IIIB/C or IV	Ivonescimab vs. Pembro	PFS, OS	PFS HR: 0.51 [0.38–0.69]
	HARMONi-A	III, active, not recruiting	Advanced NSCLC, EGFR mutant, failed EGFR-TKI treatment	Ivonescimab + CB vs. Placebo + CB	PFS	PFS HR: 0.46 [0.34–0.62]
	HARMONi-6	III, active, not recruiting	Stage IIIB/C or IV, SQ NSCLC	Ivonescimab + PC vs. Tisl + PC	PFS	PFS HR: 0.60 [0.46–0.78]
KN046 (PD-1 X CTLA-4) [62]	NCT05420220	II, recruiting	Stage IIIB/IV, untreated, received systemic therapy	KN046 + Axitinib	ORR	PD-L1 TPS ≥ 1%: ORR: 54.5 [38.8–69.6]; PD-L1 TPS ≥ 50%: ORR: 66.7 [38.4–88.2]
PM8002 (PD-1 X VEGF-A) [69]	NCT05756972	II/III, active, not recruiting	EGFR mutant, stage IIIB/IV NSQ NSCLC, failed EGFR-TKI treatment	PM8002 + Chemo vs. Chemo	ORR, PFS	ORR: 54.7 [41.8–67.2]; DCR: 95.3 [86.9–99.0]
Acasunlimab (PD-1 X 4-1BB) [70]	NCT05117242	II, active, not recruiting	Relapsed/refractory mNSCLC, after SOC with ICI	Acasunlimab vs. Acasunlimab + Pembro vs. Casunlimab + Pembro	ORR	ORR: 31 vs. 25 vs. 30; DCR: 50 vs. 65 vs. 75
Tobemstomig (PD-1 X LAG-3) [71]	NCT05775289	II, active, not recruiting	Locally advanced, untreated unresectable, mNSCLC	Tobemstomig + Chemo vs. Pembro + Chemo	ORR, PFS	ORR: 41 vs. 42

Abbreviations: CB, pemetrexed + carboplatin; CI, confidence intervals; chemo, chemotherapy; DCR, disease control rates; EGFR, Epidermal Growth Factor Receptor; HR, hazard ratio; ICI, immune checkpoint inhibitor; mNSCLC, metastatic non-small cell lung cancer; NSQ, non-squamous; NSCLC, non-small cell lung cancer; ORR, objective response rate; OS, overall survival; PC, carboplatin + paclitaxel; pembro, pembrolizumab; PFS, progression-free survival; SOC, standard of care; SQ, squamous; tisl, tislelizumab; TKI, tyrosine kinase inhibitors.

## Data Availability

No new data were created or analyzed in this study. Data sharing is not applicable to this article.

## Supplementary Materials

The following supporting information can be downloaded at https://www.mdpi.com/article/10.3390/cancers18040709/s1: Table S1: Current status of bispecific antibody development in NSCLC (Phase II and III trials status); Supplementary Material: SANRA checklist.

## Abbreviations

The following abbreviations are used in this manuscript:

ADCs	antibody-drug conjugates
ADCC	antibody-dependent cellular cytotoxicity
ADCP	antibody-dependent cellular phagocytosis
Aes	adverse events
ALK	Anaplastic Lymphoma Kinase
BBB	blood–brain barrier
BsAb	bispecific antibodies
BTB	blood–tumor barrier
chemo-IO	chemoimmunotherapy
CNS	central nervous system
CoGs	cost of goods
CRS	cytokine release syndrome
CTLA-4	Cytotoxic T-Lymphocyte-Associated protein 4
DAMPs	danger-associated molecular patterns
DCR	disease control rates
DOR	durability of response
EGFR	epidermal growth factor receptor
ICD	immunogenic cell death
ICI	immune checkpoint inhibitor
IgG	immunoglobulin G
irAEs	immune-related adverse events
iRECIST	immune-response evaluation criteria in solid tumors
IRRs	infusion-related reactions
ITIM	Immunoreceptor Tyrosine-based Inhibitory Motif
LAG-3	Lymphocyte-Activation Gene 3
MDSCs	myeloid-derived suppressor cells
MHC	major histocompatibility complex
mNSCLC	metastatic non-small cell lung cancer
ORR	overall response rates
OS	overall survival
PD-1	programmed cell death protein 1
PD-L1	programmed death-ligand 1
PFS	progression-free survival
PK	Pharmacokinetic
SANRA	scale for the quality assessment of narrative review articles
SCLC	small cell lung cancer
SoC	standard of care
TGF-β	Transforming Growth Factor-β
TIGIT	T-cell Immunoreceptor with Ig and ITIM domains
TKIs	tyrosine kinase inhibitors
TME	tumor microenvironment
TPS	tumor proportion score
