# Supplementary material for: Evolution of Immune Checkpoint Blockade in Metastatic NSCLC: A Narrative Review of Emerging Bispecific Antibodies and the Practical Challenges of Clinical Integration
Source: Cancers (Basel). 2026 Feb 22;18(4):709. doi: 10.3390/cancers18040709 (PMC12939777; doi:10.3390/cancers18040709)
Supplement: Supplementary file 1 [file cancers-18-00709-s001.zip › cancers-4126043-supplementary.pdf]

cancers

MDPI

Supplementary Materials: Evolution of Immune Checkpoint Blockade in Metastatic NSCLC: A Narrative Review of Emerging Bispecific Antibodies and the Practical Challenges of Clinical Integration

Jin Hyoung Kang

Table S1. Bispecific antibody development in NSCLC (Phase II & III trials status).

Bispecific Antibody (Targets, Reference)	Study	Phase	Eligibility	Intervention	Primary endpoint
Ivonescimab (PD-1 X VEGF) [14-17]	HARMONi	III, active, not recruiting	EGFR mutant, stage IIIB/IV	Ivonescimab+CB vs. Placebo+CB	PFS, OS
	NCT04736823	II, recruiting	Stage IIIB/C or IV	Ivonescimab+CB/PC/doce	ORR
	HARMONi-3	III, recruiting	First-line mNSCLC	Ivonescimab+Chemo vs. Pembro+Chemo	PFS, OS
	HARMONi-7	III, recruiting	First-line mNSCLC, PD-L1 TPS≥50%	Ivonescimab vs. Pembro	PFS, OS
KN046 (PD-1 X CTLA-4) [18]	NCT06020352	II/III, recruiting	Stage IB-IIB, neoadj setting	KN046+axitinib	MPR and surgical resection rate
PM8002 (PD-1 X VEGF-A) [19]	LungMate-026	II, not yet recruiting	Stage II-III, neoadj setting	Chemo+PM8002 vs. Chemo+Sintilimab	PCR rate
Acasunlimab (PD-1 X 4-1BB) [20]	ABBIL1TY NSCLC-06	III, recruiting	PD-L1 positive, mNSCLC, treated with PD-1/PD-L1 inhibitor	Acasunlimab+Pembro vs. Doce	OS
Cadonilimab (PD-1 X CTLA-4) [21-27]	NCT06467500	II, recruiting	Advanced NSCLC, received prior systemic therapy, EGFR/ALK WT	Candonilmab	ORR
	NCT06793813	II, recruiting	Stage IV NSQ NSCLC	Cadonilimab+Bevacizumab+Doce	6-month PFS
	NCT06617416	III, recruiting	Locally advanced NSCLC, unresectable	Cadonilimab vs. Sugemalimab	PFS
	LungCadX	II, recruiting	Advanced NSCLC, PD-L1 negative	Cadonilimab+Chemo	PFS
	NCT06532591	II, recruiting	Stage IB-IIIB (N2), resectable, PD-L1 negative	Cadonilimab+Chemo	MPR
	NCT06277674	II recruiting	T790M negative, advanced NSQ NSCLC, resistant to EGFR-TKI	Cadonilimab+Pemetrexed+Anlotinib	ORR
	NCT05215067	II, active, not recruiting	mNSCLC, received prior systemic therapy, EGFR/ALK WT	Candonilimab+Doce	ORR
Rilvegostomig (PD-1 X TIGIT) [28,29]	ARTEMIDE-Lung04	III, recruiting	First-line treatment of PD-L1 high mNSCLC	Rilvegostomig vs. Pembro	PFS, OS
	ARTEMIDE-Lung02	III, recruiting	First-line treatment of SQ mNSCLC, PD-L1≥1%	Rilvegostomig+Chemo vs. Pembro+Chemo	PFS, OS

 https://doi.org/10.3390/cancers18040709

IMM2510 (PD-L1 X VEGF) [30]	NCT06746870	II, not recruiting	Stage IV recurrent/mNSCLC, EGFR WT, negative for ALK/ROS1 fusion genes, untreated	IMM2510 vs. Chemo (CB/CP for NSQ NSCLC, Cpa/PC for NSQ NSCLC)	ORR
Volrustomig (PD-1 X CTLA-4) [31]	eVOLVE-Lung02	III, recruiting	mNSCLC, lack activating EGFR/ALK/ROS1	Volrustomig+CB/PC vs. Pembro+CB/PC	PFS, OS
SSGJ-707 (PD-1 X VEGF) [32]	NCT06412471	II, recruiting	Advanced NSCLC	SSGC-707+CB/PC	ORR, safety and tolerability

Abbreviations: ALK, Anaplastic Lymphoma Kinase; CB, pemetrexed + carboplatin; CI, confidence intervals; chemo, chemotherapy; CP, cisplatin + pemetrexed; CPa, cisplatin +paclitaxel; doce, docetaxel; EGFR, Epidermal Growth Factor Receptor; HR, hazard ratio; HR, hazard ratio; ICI, immune checkpoint inhibitpr; mNSCLC, metastatic non-small cell lung cancer; MPR, major pathologic response; neoadj, neoadjuvant; NSCLC, non-small cell lung cancer; NSQ, non-squamous; ORR, objective response rate; OS, overall survival; PC, carboplatin + paclitaxel; PCR, pathological complete response; PD-1, programmed cell death protein 1; PD-L1, programmed death-liagand 1; pembro, pembrolizumab; PFS, progression-free survival; SOC, standard of care; SQ, squamous; TPS, tumor proportion score; WT, wild-type.

https://doi.org/10.3390/cancers18040709
